# New records of bats and terrestrial small mammals from the Seli River in Sierra Leone before the construction of a hydroelectric dam

**DOI:** 10.3897/BDJ.7.e34754

**Published:** 2019-06-18

**Authors:** Natalie Weber, Ricarda Wistuba, Jonas J Astrin, Jan Decher

**Affiliations:** 1 Independent Research Consultant, Fuerth, Germany Independent Research Consultant Fuerth Germany; 2 ZFMK, Bonn, Germany ZFMK Bonn Germany

**Keywords:** Bumbuna Phase II hydroelectric project, Upper Guinean Forests, Chiroptera, Rodentia, Soricomorpha

## Abstract

Sierra Leone is situated at the western edge of the Upper Guinean Forests in West Africa, a recognised biodiversity hotspot which is increasingly threatened by habitat degradation and loss through anthropogenic impacts. The small mammal fauna of Sierra Leone is poorly documented, although bats and rodents account for the majority of mammalian diversity. Based on morphological, genetic and echolocation data, we recorded 30 bat (Chiroptera), three shrew (Soricomorpha) and eleven rodent (Rodentia) species at the Seli River in the north of the country in 2014 and 2016, during a baseline study for the Bumbuna Phase II hydroelectric project. In 2016, 15 bat species were additionally documented at the western fringe of the Loma Mountains, a recently established national park and biodiversity offset for the Bumbuna Phase I dam. Three bat species were recorded for the first time in Sierra Leone, raising the total number for the country to 61. Further, two bat species are threatened and endemic to the Upper Guinean Forest and several taxa of small mammals are poorly known or represent undescribed species. Overall, the habitats of the project area supported a species-rich small mammal fauna including species of global conservation concern. Suitable mitigation measures and/or offsets are necessary to maintain biodiversity and ecosystems in a region that is under high human pressure.

## Introduction

Sierra Leone is situated at the western edge of the Upper Guinean Forest biodiversity hotspot in West Africa (Myers et al. 2000, Bakarr et al. 2001, Bakarr et al. 2004). Data on species distribution and ecology is disproportionately scarce compared to countries further east in the hotspot such as Ghana (Luiselli et al. 2019), in particular for small mammals. The most comprehensive review of Sierra Leone mammals is an annotated checklist of Grubb et al. (1998) and only one survey of bats and terrestrial small mammals has been published since then (Decher et al. 2010). As per this study, 58 species of bats, 10 species of shrews and 40 species of rodents (excluding squirrels) were known to occur in the country. In general, bats and rodents constitute the most species-rich groups of mammals. Together with shrews, the three groups have important ecological roles and provide various ecosystem services (e.g. insect control, seed dispersal, pollination). Many species in these groups have highly specific habitat requirements and bats are recognised as a particularly suitable indicator group to assess changes in the condition of ecosystems and conservation priorities (Jones et al. 2009). Beyond, cryptic diversity is high in all three study groups and further research is needed to define species boundaries of respective taxa and resolve taxonomic questions. Updates on distribution and ecological data for small mammals in Sierra Leone are thus urgently needed to identify conservation priorities and manage human impacts on biodiversity. Besides agricultural expansion, these impacts include large-scale development projects such as hydroelectric dams and mines, with effects on West African biodiversity that need to be thoroughly assessed, monitored and mitigated. In addition, species distribution and ecological data are essential as a base for a range of other studies, e.g. zoonotic disease research (Guyton and Brook 2015, Han et al. 2016, Pigott et al. 2016).

Within the Upper Guinean Forests, particularly the few (sub-)montane areas represent centres of species richness and are highly significant for the conservation of biodiversity (Bakarr et al. 2001, Bakarr et al. 2004, Burgess et al. 2004, Carr et al. 2015). Our survey was conducted in the Bumbuna Phase II area (hereafter: Bumbuna II) at the Seli River at the foothills of the Sula Mountains in the Northern Province. This part of the Northern Province is marked by mountainous elements in an otherwise eroded landscape. Bumbuna II is characterised by a mountainous forest-savannah mosaic, with forest patches along water-bodies and in steep ravines, as well as farmbush and agricultural lands. Bats were additionally sampled at the western outskirts of the Loma Mountains National Park (hereafter: Loma). The Loma Mountains are a recognised Key Biodiversity Area in the country (Carr et al. 2015) and were designated as a national park to offset impacts of the Bumbuna Phase I hydroelectric project completed in 2009 (Forestry Division 2012), yet records of bats from the area are only sporadic and dated (Atkinson et al. 1996, Grubb et al. 1998). Both study areas are situated within or adjacent to a region classified as exceptionally important for the conservation of mammals within the Upper Guinean Forests (Bakarr et al. 2001) and within a continental hotspot of endemism for bat species according to a high-resolution model by Herkt et al. (2016).

This study provides new distribution data for bats and terrestrial small mammals from mountainous areas in northern Sierra Leone in a regional context. We present updates on the distribution of several range-restricted species and the national species list. Our data constitute the baseline to assess impacts on bats and terrestrial small mammals from the development of the Bumbuna Phase II hydroelectric dam, and to identify habitats and species of conservation concern for appropriate environmental management.

## Material and methods

### Study area

Bumbuna II is located along the Seli River, approximately 25 km south-southwest of Kabala, the capital of Koinadugu District (Fig. 1) and 32 km upstream of the existing Bumbuna Phase I dam. It includes a section of the river of approximately 25 km, meandering in an east-west direction, and the transmission line connecting the Bumbuna Phase I area (hereafter: Bumbuna I; Decher et al. 2010) and Bumbuna II. The projected reservoir area covers a surface of 115 km^2^. Field work was conducted between 21 May and 4 June 2014 during the early wet season. Bats were sampled a second time between 24 March and 8 April 2016 towards the end of the dry season. Mean annual rainfall in the area is above 2,500 mm with a single wet season from May to November and a mean monthly temperature of 25.7°C (WorldClim 2.5' grid, Hijmans et al. 2005).

We sampled bats and terrestrial small mammals to cover all major habitat types in the study area (Table 1). Study sites were situated in riparian forests along the Seli River and its tributaries, wooded savannah, managed forest patches, grassland, farmbush, rocky outcrops and agricultural lands (slash-and-burn farming, pasture lands, oil palm plantations). Such highly heterogeneous mosaic landscapes are characteristic for the Guinean forest-savannah ecoregion, which is situated between the lowland rainforest zone to the south and the Sudanian savannahs to the north (Kelman and Burgess 2001). The study area in Loma is situated roughly 40 km east-southeast from Bumbuna II. The Loma Mountains are the source area of many tributaries of major rivers, e.g. the Niger River (Lebbie 2001), contain the largest tract of montane forest in Sierra Leone, and rise up to the highest peak in West Africa west of Mount Cameroon, Mount Bintumani (1,947 m asl). The study area comprised a small river surrounded by a mixture of forest, swamp and farmbush.

### Capture and sampling

We employed 12 m and 6 m mist nets (Ecotone, Poland: 2.8 m height, 5 shelves, 16 mm mesh, 2x70 dernier netting) set at both ground and canopy level and a three-bank harp trap (Austbat, FaunaTech, Australia) to capture bats, following international standard methods (Kunz and Parsons 2009). Nets were opportunistically placed at and across rivers and streams, forest edges, footpaths or other presumed flyways to maximise capture success. All nets and the harp trap were opened at sunset around 1845 h, checked every 30-60 minutes, and usually closed before 0100 h. Overall sampling effort with nets near ground level was 330.4 net hours, calculated as 12 m mist net equivalent, 117.3 net hours with canopy nets and 47.7 harp trap hours. We also visited three accessible caves which were reported to harbour bats. Coordinates of all study sites were recorded with a hand-held GPS receiver (Garmin 62s; Table 1).

Captured bats were individually kept in cloth bags until processing. We took body mass and forearm length of all individuals and additional standard measurements of collected specimen (head and body, tail, ear, hind foot). Measurements were taken with a dial caliper (Holex 150/01, Germany) to the nearest 0.1 mm. Body mass was measured with spring balances (Pesola 50 g, 100 g, 600 g, Switzerland). Sex, age class and reproductive state were determined visually. Echolocation calls of representatives of hipposiderid and rhinolophid bats were recorded with a Pettersson D240x bat detector in 10x time expansion mode and transferred to a H2 digital sound recorder (www.zoom.co.jp). The constant-frequency (cf) component of echolocation calls was measured to aid identification of problematic taxa, as it is highly specific in many hipposiderid and rhinolophid bats. Most calls were obtained from hand-held bats and on one occasion of bats in flight in a cave. Calls were later analysed with the software BatSound (Version 1.3.3). A total of 18 voucher specimens of species not previously recorded in the area or country was preserved in 70% ethanol for documentation and tissue samples were preserved in 95% ethanol. Additional genetic samples were collected from the patagium of some released individuals. Specimens and tissue samples are deposited in the research collection of Jakob Fahr (RCJF; Division of Evolutionary Biology, TU Braunschweig, Germany). Collection numbers have not yet been assigned, but collection plus field number represents a unique identifier. In addition to published records of bats from Sierra Leone, the exhaustive database AfriBats (http://afribats.myspecies.info) provided context for our results, including museum records from: National History Museum, London (BMNH); Musée Royal de l’Afrique Centrale, Tervuren (MRAC); Royal Ontario Museum, Toronto (ROM); US National Museum, Washington D.C. (USNM).

For terrestrial small mammals, each of six sites was sampled for one to three consecutive nights. We mainly used Sherman live traps (LFA Life Trap, H.P. Sherman Traps, Inc.). Some Victor Metal Pedal Rat snap traps were set out in places difficult to access, e.g. on tree branches and vines, rock outcrops or in creek beds and to capture trap-shy species. At each site, one or two pitfall lines of 40 metres of plastic drift fence with four or five buckets were installed. Four Tomahawk live traps (203 Collapsible Squirrel Life Trap, Tomahawk Live Traps) were used opportunistically to target larger species. Palm fruit, peanut butter with oats and cat food was used as bait. Visual estimations of the microhabitat (canopy and ground cover, see Suppl. material 1) of each captured terrestrial mammal in one square metre centred on each trap were recorded on standardised habitat data sheets. All coordinates were recorded using a Garmin eTrex GPS receiver (Table 1). The body mass of each captured individual was measured with spring balances (Pesola 10g, 30g, 50 g, 100 g, 1,200 g, Switzerland). Sex and age class were determined visually. Voucher specimens (n=49) were conserved in 70% ethanol either as a whole specimen or as skull and skin. All shrews were kept due to the difficulty in identifying West African shrews in the field. Tissue samples from liver, kidney and spleen were conserved in 96% ethanol. All material is housed at the Zoological Research Museum Alexander Koenig (ZFMK), Bonn, Germany. If possible, measurements of hind foot and tail length were also taken from live animals.

Survey techniques complied with international standard methods for measuring and monitoring small mammal diversity (Voss and Emmons 1996, Kunz and Parsons 2009) and guidelines approved by the American Society of Mammalogists (Sikes and Mammalogists of the Animal Care Use Committee of the American Society 2016). Identification in the field was aided by Rosevear (1965) and our own previously collected data for bats and by Rosevear (1969), Meester and Setzer (1971), Kingdon (1997) for small terrestrial mammals. Taxomomy and nomenclature follow Simmons (2005) for bats and Hutterer (2005) as well as Musser and Carleton (2005) for terrestrial mammals, unless subsequent taxonomic updates were available. The IUCN Red List status follows the latest update 2018.2 (IUCN 2019).

### Molecular phylogenetic and distance-based analyses

**Figure 2.**
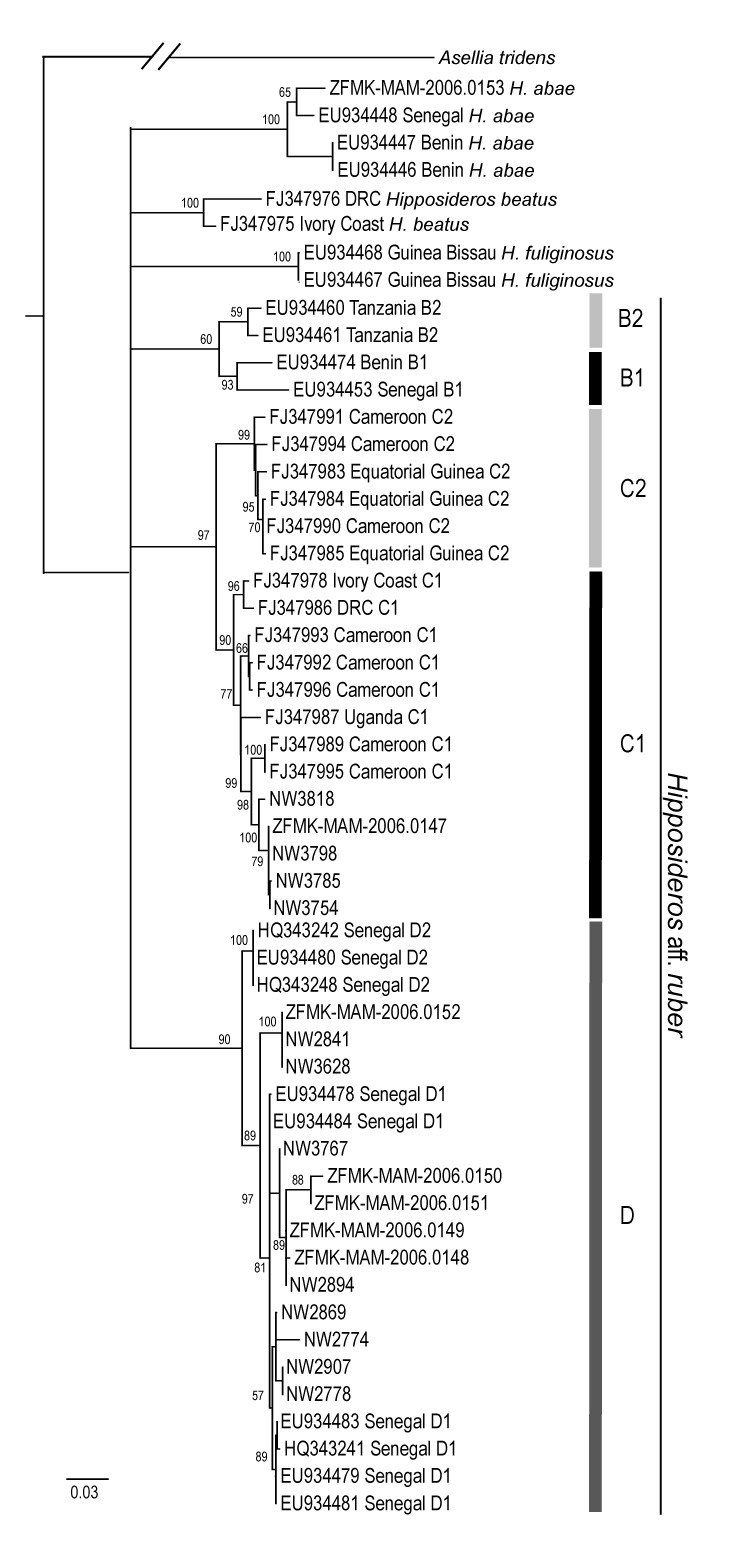
Maximum likelihood tree showing phylogenetic relationship of *Hipposideros* bats (in particular within the H. aff. ruber species complex) from the Bumbuna Phase II and I areas, and from other localities in Africa (Vallo et al. 2008, Vallo et al. 2011), based on cytochrome b (Cyt-*b*). Individuals of H. aff. ruber from Bumbuna II are marked with field numbers (NWxxxx). Scale bar indicates mean number of nucleotide substitutions per site. Bootstrap values are shown next to the corresponding nodes. Vertical bars denote the different Hipposideros aff. ruber lineages.

The *Hipposideros ruber*-*caffer* species complex (Chiroptera: Hipposideridae) comprises several distinct lineages, which likely represent cryptic bat species (Vallo et al. 2008). Genetic samples collected from the patagium of 12 released bats belonging to this group were analysed and present the first respective effort for individuals from Sierra Leone. The analyses also included six individuals of H. aff. ruber collected in Bumbuna I (Decher et al. 2010). All these samples are deposited at the Zoological Research Museum Alexander Koenig (ZFMK), Bonn, Germany and sequences are in GenBank (https://www.ncbi.nlm.nih.gov/genbank/). Genomic DNA was isolated using the Blood and Tissue kit by Qiagen (Hilden, Germany). DNA extracts are available from the ZFMK Biobank, Bonn. Following Vallo et al. (2008), we amplified the complete mitochondrial cytochrome b (Cyt-*b*) gene by using universal primers L14724 and H15915 (Irwin et al. 1991). This combination worked in only six of 19 cases (producing fragments of ca. 1100 bp). For the remaining specimens, we combined the primers Molcit-F (Ibáñez et al. 2006) and MVZ16 (Smith and Patton 1993), yielding a ca. 800 bp PCR product. Using the Qiagen Multiplex kit, PCRs were run on a GeneAmp PCR System 2700 (Life Technologies, Carlsbad, CA, USA), following a touchdown protocol (55 to 40°C, then 20 cycles at 40°C). Following enzymatic clean-up, double-stranded sequencing was conducted on an automated ABI 3730XL sequencer (Applied Biosystems) at the Macrogen facility, Amsterdam, NL. Sequences were assembled and inspected using Geneious R7 (Biomatters, Auckland, New Zealand). Sequence alignment used the MUSCLE algorithm (Edgar 2004). To focus on phylogenetic relationships within the genus *Hipposideros*, a Maximum Likelihood tree was inferred with RAxML-HPC vers. 8.1.24 (Stamatakis 2014). We used 19 of our own and 35 previously published sequences (Fig. 2; Vallo et al. 2008, Vallo et al. 2011). The trident leaf-nosed bat, *Asellia tridens*, functioned as outgroup. A GTR+Γ model was applied following the programme recommendations. The dataset was partitioned to treat 3rd codon positions separately from 1st and 2nd positions. The analysis used the "-f a" option (bootstrap analysis and search for best-scoring ML tree in one programme run) and included 10,000 bootstrap replicates. Nodes with a bootstrap support of 50 or below were collapsed.

**Figure 3.**
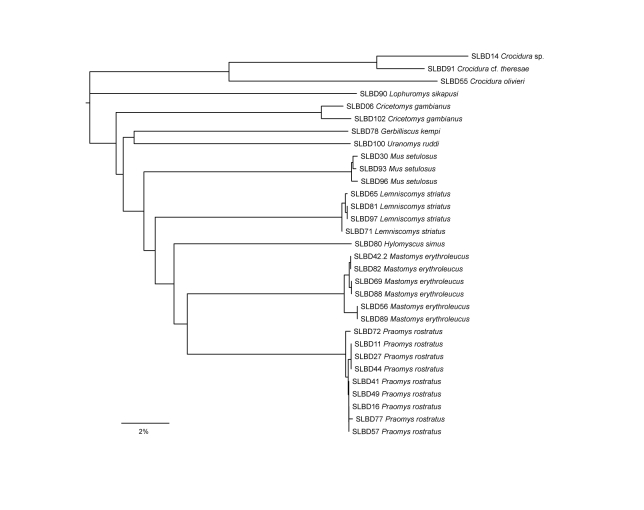
Neighbour joining tree of terrestrial small mammals from the Bumbuna Phase II area, based on *p*-distances of the DNA barcoding gene cytochrome oxidase 1 (CO1), scale bar: 2% *p*-distance.

We used genetic distances as a mean to verify identification of collected terrestrial small mammals. To that end, we obtained 35 tissue samples (mostly liver) for DNA analysis. Laboratory protocol and DNA sample deposition are identical to those stated above. For PCR of the cytochrome oxidase 1 (CO1) gene (DNA barcoding fragment, 658 bp), we combined the primers LCO1490-JJ and HCO2198-JJ (Astrin and Stüben 2008). Primer annealing temperatures for touchdown PCR started at 60°C and decreased to 45°C. As the purpose of this dataset was purely for specimen identification, and as the sequences came from a range of different taxa (instead of targeted phylogenetic taxon sampling), sequences were analysed using the Neighbour Joining method (Saitou and Nei 1987), implemented in Geneious R7, with genetic distances shown as *p*-distances, i.e. as the proportion of diverging nucleotide sites (Fig. 3). As for Cyt-*b* in bats, the MUSCLE algorithm was used for alignment.

### Statistic analyses

Smoothed species accumulation curves were generated with the programme EstimateS (Version 9.1; Colwell 2013). The sample-based rarefaction curve was calculated with the Mao Tau function (Colwell et al. 2004) and the graphs were rescaled by individuals. We used three non-parametric incidence-based estimators (ICE: Incidence-based coverage estimator, Jackknife 1 and Jackknife 2) to extrapolate the expected numbers of species occurring in Bumbuna II from our samples (Colwell 2013, Gotelli and Colwell 2011).

## Data resources

### Bats

Voucher specimens

Bumbuna II: *Rhinolophus landeri* (RCJF-NW2811); *Hipposideros marisae* (RCJF-NW2812); *Macronycteris vittatus* (RCJF-NW3664); *Nycteris macrotis* (RCJF-NW2823); *Nycteris thebaica* (RCJF-NW2808); *Coleura afra* (RCJF-NW3790); *Pipistrellus nanulus* (RCJF-NW2891, RCJF-NW2904); *Scotoecus hirundo* (RCJF-NW2900); *Scotophilus viridis* (RCJF-NW2855); *Scotophilus dinganii* (RCJF-NW2859); *Glauconycteris poensis* (RCJF-NW2866); *Mops condylurus* (RCJF-NW2851). Loma: *Scotophilus nux* (RCJF-NW3697); *Chaerephon nigeriae* (RCJF-NW3682); *Mops nanulus* (RCJF-NW3645); *Mops thersites* (RCJF-NW3662); *Mops trevori* (RCJF-NW3646).

DNA voucher and GenBank accession numbers

Bumbuna II: Hipposideros aff. ruber C1 (ZFMK-DNA-FC19476444, MH713747; ZFMK-DNA-FC19476428, MH713749; ZFMK-DNA-FC19476460, MH713750; ZFMK-DNA-FC19476452, MH713751); Hipposideros aff. ruber D(1) (ZFMK-DNA-FC19476467, MH713740; ZFMK-DNA-FC19476396, MH713741; ZFMK-DNA-FC19476404, MH713743; ZFMK-DNA-FC19476412, MH713744; ZFMK-DNA-FC19476420, MH713745; ZFMK-DNA-FC19476468, MH713748); Hipposideros aff. ruber D(?) (ZFMK-DNA-FC19476380, MH713742). Loma: Hipposideros aff. ruber D(?) (ZFMK-DNA-FC19476436, MH713746).

### Terrestrial small mammals

Voucher specimens

Bumbuna II: *Crocidura olivieri* (ZFMK-MAM-2014.0601); Crocidura cf. theresae (ZFMK-MAM-2014.0603); *Crocidura* sp.1 (ZFMK-MAM-2014.0602); *Cricetomys gambianus* (ZFMK-MAM-2014.0641; ZFMK-MAM-2014.0642; ZFMK-MAM-2014.0643); *Gerbilliscus kempi* (ZFMK-MAM-2014.0604); *Lophuromys sikapusi* (ZFMK-MAM-2014.0610); *Uranomys ruddi* (ZFMK-MAM-2014.0640); *Hylomyscus simus* (ZFMK-MAM-2014.0614); *Lemniscomys striatus* (ZFMK-MAM-2014.0607‒0609; ZFMK-MAM-2014.0638‒0639; ZFMK-MAM-2014.0644); *Malacomys edwardsi* (ZFMK-MAM-2014.0611‒0612); *Mastomys erythroleucus* (ZFMK-MAM-2014.0632; ZFMK-MAM-2014.0634‒0636; ZFMK-MAM-2014.0646; ZFMK-MAM-2014.0685); *Mus musculoides*/*minutoides* (ZFMK-MAM-2014.0613); *Mus setulosus* (ZFMK-MAM-2014.0615‒0616); *Praomys rostratus* (ZFMK-MAM-2014.0605‒0606; ZFMK-MAM-2014.0617‒0631; ZFMK-MAM-2014.0633; ZFMK-MAM-2014.0637; ZFMK-MAM-2014.0645).

DNA voucher and GenBank accession numbers

Bumbuna II: *Crocidura olivieri* (ZFMK-TIS-24165, ZFMK-DNA-0171606048, MH713719); Crocidura cf. theresae (ZFMK-TIS-26117; ZFMK-DNA-0171661915; MH713734); *Crocidura* sp.1 (ZFMK-TIS-24084, ZFMK-DNA-0171606069, MH713711); *Cricetomys gambianus* (ZFMK-TIS-24076, ZFMK-DNA-0171606071, MH713709; ZFMK-TIS-26466, ZFMK-DNA-0171661910, MH713739); *Gerbilliscus kempi* (ZFMK-TIS-26099, ZFMK-DNA-0171606057, MH713727); *Lophuromys sikapusi* (ZFMK-TIS-26115, ZFMK-DNA-0171661916, MH713733); *Uranomys ruddi* (ZFMK-TIS-26464, ZFMK-DNA-0171661911, MH713738); *Hylomyscus simus* (ZFMK-TIS-26101, ZFMK-DNA-0171606058, MH713728); *Lemniscomys striatus* (ZFMK-TIS-24177, ZFMK-DNA-0171606052, MH713722; ZFMK-TIS-24187, ZFMK-DNA-0171606054, MH713724; ZFMK-TIS-26103, ZFMK-DNA-0171606059, MH713729; ZFMK-TIS-26462, ZFMK-DNA-0171661912, MH713737); *Mastomys erythroleucus* (ZFMK-TIS-24154, ZFMK-DNA-0171606064, MH713716; ZFMK-TIS-24167, ZFMK-DNA-0171606049, MH713720; ZFMK-TIS-24185, ZFMK-DNA-0171606053, MH713723; ZFMK-TIS-26105, ZFMK-DNA-0171661919, MH713730; ZFMK-TIS-26111, ZFMK-DNA-0171661918, MH713731; ZFMK-TIS-26113, ZFMK-DNA-0171661917, MH713732); *Mus setulosus* (ZFMK-TIS-24148, ZFMK-DNA-0171606066, MH713714; ZFMK-TIS-26362, ZFMK-DNA-0171661914, MH713735); *Praomys rostratus* (ZFMK-TIS-24088, ZFMK-DNA-0171606068, MH713712; ZFMK-TIS-24144, ZFMK-DNA-0171606067, MH713713; ZFMK-TIS-24150, ZFMK-DNA-0171606065, MH713715; ZFMK-TIS-24156, ZFMK-DNA-0171606063, MH713717; ZFMK-TIS-24160, ZFMK-DNA-0171606061, MH713718; ZFMK-TIS-24169, ZFMK-DNA-0171606050, MH713721; ZFMK-TIS-26095, ZFMK-DNA-0171606055; ZFMK-TIS-26097, ZFMK-DNA-0171606056, MH713726; ZFMK-TIS-24080, ZFMK-DNA-0171606070, MH713710).

## Results

### Bats

In total, we captured 352 bats (Bumbuna II: 268, Loma: 84) in 34 species (Bumbuna II: 29, Loma: 14) and eight families (Tables 2, 3), including three first country records. One additional species (*Rhinolophus fumigatus*) was acoustically recorded in Bumbuna II. Overall capture success was 0.73 bats per 12 m net hour (b/nh) with mist nets at ground level, 0.70 b/nh with canopy nets and 0.44 bats per trap hour (b/th) with the harp trap. Of 135 bats captured in Bumbuna II in 2014, 25 bats in three species were fruit bats (18.5%). In 2016, the 133 individuals recorded in Bumbuna II included 112 fruit bats in eight species (84.2%). Almost half of the species occurring in Bumbuna II are (mainly) associated with forest habitats (n = 13, 43.3%), with 36.7% (n = 11) of the species predominantly inhabiting savannahs and 20.0% (n = 6) equally using savannahs and forests (Table 2). In Loma, more than half of the species depend on forest habitats (n = 8, 53.3%) and slightly more than one quarter (n = 4, 26.7%) occurs predominantly in savannahs, while 20.0% (n = 3) are found in forests and in savannahs. Thirteen species (43.3%) in Bumbuna II depend at least partially on caves or cave-like structures as day roost (Table 2). The percentage was slightly higher when including records from the wider area (n = 17, 45.9%) and lower in Loma (n = 4, 26.7%). The species accumulation curves for bats from Bumbuna II in 2014, 2016, and combined for both years, do not yet approach an asymptotic plateau, indicating that the bat inventory of the area is not complete (Fig. 4). Based on data from both study years combined, the total number of bat species occurring in Bumbuna II was estimated to be 38.3 (ICE), 39.4 (Jackknife1) and 45.0 (Jackknife2), respectively.

### Terrestrial small mammals

**Figure 5.**
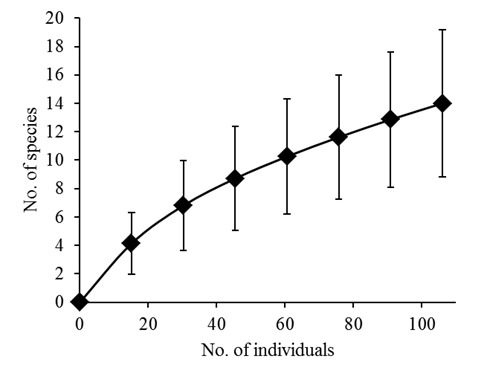
Sample-based species accumulation curve for terrestrial small mammals in the Bumbuna Phase II area in 2014, rescaled by individuals (see Suppl. material 3). Vertical bars: ± 1 SD.

In 1,423 trap nights, 106 individuals belonging to 14 species, comprising three shrew and eleven rodent species, were captured (Tables 2, 4). Additionally, two squirrel species were observed and identified, but not included in the standardised statistic analyses. Our study adds six species to the records from Bumbuna I. Overall trapping success was 7.4% with a range from 2.9% to 12.8% (Table 4). *Praomys rostratus* was the most frequently found species with 67.0%, followed by *Cricetomys gambianus, Lemniscomys striatus* and *Mastomys erythroleucus* with 6.6% each. Almost half of the species (46.2%) depend on savannah habitat, 23.1% constitute forest species and 30.8% are known to occur in both habitat types. The species accumulation curve shows a steady increase and no levelling off (Fig. 5). The richness estimators predicted 21 to 27 species (ICE: 26.7, Jacknife1: 20.9, Jackknife2: 25.4). The genetic analyses supported most species identifications, but the assignment of two shrews remains uncertain (Fig. 3).

### Checklist

In the following species accounts, we present information on systematics, distribution and conservation status of new species records for the wider Bumbuna area and Loma in 2014 and 2016. For species already documented from Bumbuna I (Decher et al. 2010) and encountered in our study, we only list new information. Species only found in Bumbuna I and previous records from within a radius of 40 km (Suppl. material 4) are included in Table 2. If not otherwise mentioned, females did not show signs of reproductive activity.

**Order Chiroptera**

**Family Pteropodidae**

***Micropteropus pusillus* (Peters, 1868)** Peters's Dwarf Epauletted Fruit Bat

Eight individuals were captured in Bumbuna II in savannah habitats in 2014 (B3), and 19 individuals at the same site and along rivers in 2016 (B3, B7, B8). Out of the 27 individuals, 17 were females. Two females were pregnant (30 + 31 Mar) and five were lactating (6 + 7 Apr, 28 May).

***Epomops buettikoferi* (Matschie, 1899)** Buettikofer's Epauletted Fruit Bat

With 49 individuals (Bumbuna II: 45, Loma: 4), this was the second most frequently encountered fruit bat species in our study. In Bumbuna II, the species was found at five sites (B3, B4, B6, B7, B8) in savannah and riparian forest habitats in varying condition. We captured 19 females, including one pregnant (30 Mar) and six lactating (24, 30, 31 Mar, 1 + 7 Apr) females.

***Eidolon helvum* (Kerr, 1792)** African Straw-coloured Fruit Bat

Four individuals of this species were captured, two in Bumbuna II (B7) and two in Loma, representing new records for both areas. All four individuals were encountered between agricultural lands and riparian forests. *Eidolon helvum* is known from several other localities, mostly day roosts, across the country (Grubb et al. 1998). The species often forms large colonies comprising up to several thousand or millions of individuals. In West Africa, colonies are frequently situated in cities, in Sierra Leone e.g. in Freetown and Makeni (N. Weber pers. observation). *Eidolon helvum* is migratory and its presence in Bumbuna II and Loma is probably seasonal. It is ranked as “Near Threatened” by the IUCN Red List (IUCN 2019) and included on Appendix II of the Convention for the Conservation of Migratory Species of Wild Animals (CMS). This fruit bat is threatened by unsustainable hunting throughout its range, as has been examined, for instance, in Ghana (Kamins et al. 2011).

***Hypsignathus monstrosus* H. Allen, 1861** Hammer-headed Fruit Bat

Male individuals of this largest African bat were heard calling in Loma and one sub-adult female was captured with a canopy net in Bumbuna II in 2016 (B7). *Hypsignathus monstrosus* was known from the Loma Mountains prior to this study (Atkinson et al. 1996, Grubb et al. 1998), but not from Bumbuna. The species is usually encountered in forest habitats.

***Rousettus aegyptiacus* (E. Geoffroy, 1810)** Egyptian Fruit Bat

We captured 17 individuals of the Egyptian fruit bat in our study in 2016 (B4, B8: 5, Lo1: 12). One of eight females was lactating (7 Apr). We further observed a few individuals of this fruit bat at the entrance of a cavity in Sadia Konkoma (Loa) by day. A local guide reported that the majority of individuals disappeared shortly before our visit, probably due to a recent fire which burnt much of the vegetation on the rocky slope. Another approximately 60 individuals were counted in Yafarama cave (Bb). The species is hunted for bushmeat at day roosts in the area.

***Myonycteris leptodon* K. Andersen, 1908** Sierra Leone Collared Fruit Bat

We encountered six individuals of *M. leptodon* at three sites in Bumbuna II (B1, B3, B8). Three of them were females, including one which was lactating (7 Apr).

***Myonycteris angolensis smithii* (Thomas, 1908)** Angolan Fruit Bat

Five females of this fruit bat were recorded in Loma and one male in hilly woodland savannah in Bumbuna II (B3). Two females were lactating (25 Mar). The few previous records from Sierra Leone are patchily distributed, and include the type specimen for this taxon (Grubb et al. 1998). *Myonycteris angolensis smithii* uses particular caves and hollow trees as day roost and might be restricted to mountainous forest habitats.

***Nanonycteris veldkampii* (Jentink, 1888)** Veldkamp's Epauletted Fruit Bat

With a total of 94 individuals, this species constituted the most numerous species in our study, all of them recorded in 2016. Veldkamp's bat was common in Loma (n = 44 individuals) and occurred at four sites in Bumbuna II (n = 50 individuals; B3, B4, B7, B8). Females were represented by 47 individuals, 28 being adult. Out of these, five were pregnant (25, 30, 31 Mar, 2 Apr) and eleven lactating (25, 27, 30, 31 Mar, 1, 3, 7, 8 Apr). The species was likely absent from the study area in 2014.

**Family Rhinolophidae**

***Rhinolophus landeri* Martin, 1838** Lander's Horseshoe Bat

Two males of *R. landeri* were captured on rocky slopes (B1) in 2014. Forearm length (43.4-43.9 mm) and cf frequency were in the typical range of this species (Table 5; Happold 2013a). We observed one individual emerging from an underground cave at B1 shortly after sunset, indicating a day roost of this horseshoe bat (21 May). *Rhinolophus landeri* generally depends on caves or similar structures as roosting habitat. It is distributed in much of sub-Saharan Africa and occurs mainly in savannahs and riparian forests with rocky features. The species was previously known from few records in Sierra Leone, including the Loma Mountains (Grubb et al. 1998).

***Rhinolophus guineensis* Eisentraut, 1960** Guinean Horseshoe Bat

One pregnant female was recorded in riparian forest at the Yogoron River (B7) in 2016 (30 Mar). Forearm length (46.8 mm) and echolocation frequency of this individual fit with previous observations (Table 5; Fahr 2013a). *Rhinolophus guineensis* is listed as “Vulnerable” by the IUCN Red List (IUCN 2019).

***Rhinolophus fumigatus* Sanborn, 1939** Rüppell's Horseshoe Bat

This horseshoe bat was tentatively identified by echolocation recordings from Yafarama cave (Bb; Table 5; Cotterill and Happold 2013) and the relatively large size of individuals observed in flight. It was sharing this day roost with Hipposideros aff. ruber and *Rousettus aegyptiacus*.

**Family Hipposideridae**

***Hipposideros marisae* Aellen, 1954** Aellen’s Leaf-nosed Bat

**Figure 6.**
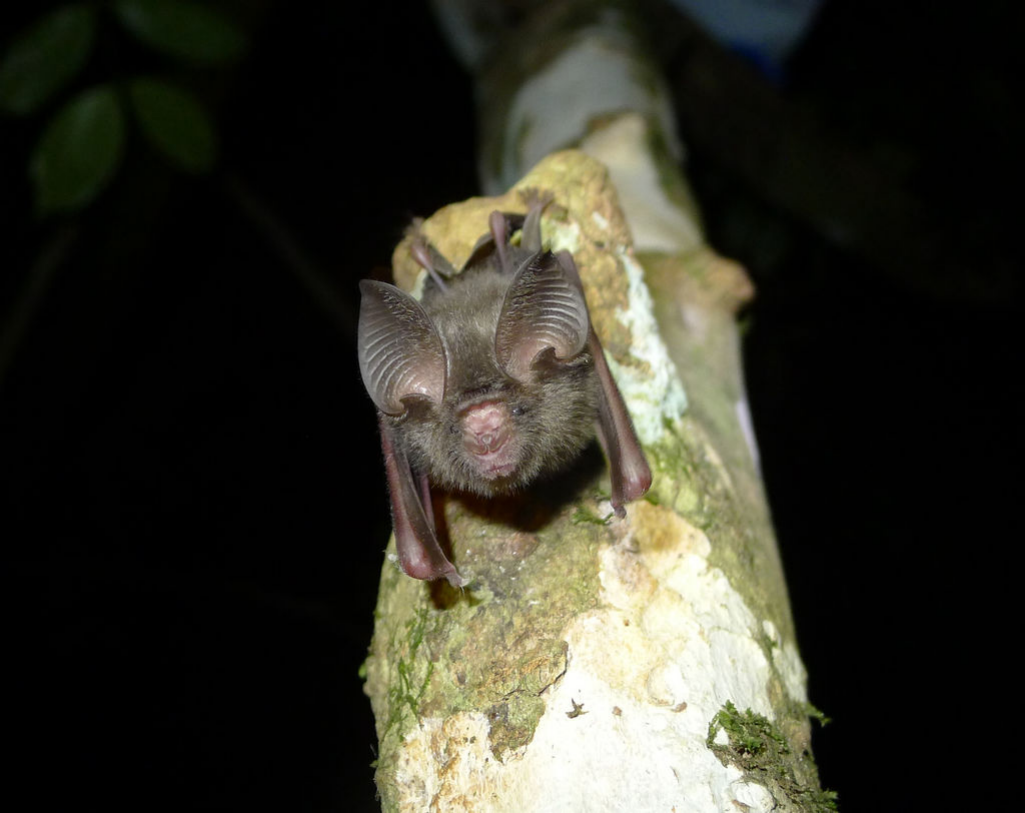
Sub-adult *Hipposideros marisae* (NW2790) recorded in the Bumbuna Phase II area in the Northern Province, Sierra Leone.

In 2014, five individuals of this small leaf-nosed bat (Fig. 6) were recorded in riparian forest at B1, where they emerged from rock cavities underneath a slope. A sixth individual was captured at a stagnant pool surrounded by *Raphia* palms at B3. The three females were lactating (21, 22, 28 May). Echolocation call frequencies of two males and two females of Aellen’s leaf-nosed bat match previous recordings of this species from Liberian Mount Nimba (Table 5; Monadjem et al. 2013).

This constitutes the first record of *H. marisae* for Sierra Leone and only the ninth observation of this species in total, with seven records dating back to at least 1990 (Koopman et al. 1995, Fahr 2013b, Monadjem et al. 2016). The Wonegizi Mountains in northwest Liberia constitute the nearest documented location for *H. marisae* (Koopman et al. 1995), at a distance of more than 250 km from our study area. Prior to this study, this species was only known from Liberia, Côte d'Ivoire and Guinea (Fahr 2013b, Monadjem et al. 2016). *Hipposideros marisae* is ranked as “Vulnerable” by the IUCN Red List (IUCN 2019). Evidence suggests that *H. marisae* is extremely rare within a restricted range, likely as it depends on suitable caves as day roost and forest habitats. Major threats to this species comprise loss of foraging and roosting habitat through deforestation, mining and other anthropogenic activities. Despite re-visiting the same localities as in 2014, the species was not encountered in the 2016 survey.

**Hipposideros aff. ruber (Noack, 1893)** Noack's Roundleaf Bat

Hipposideros aff. ruber belongs to the wider *H. caffer/ruber* species complex, which shows a high level of cryptic diversity based on Cyt-*b* (Vallo et al. 2008, Vallo et al. 2011, Hauslaib-Haidn 2011, Monadjem et al. 2013). Bats belonging to this species group were encountered at all study sites in Bumbuna II (2014: n = 68, 2016: n = 13) including the two caves and in Loma (n = 1), resulting in a total of 82 captures. High variation in forearm length and echolocation call frequency (Table 5) indicated that more than one genetic lineage was involved in our samples, but species identification with morphology and echolocation alone is currently not possible in this taxon. Following Vallo et al. (2008), analyses of mitochondrial Cyt-*b* sequences revealed that at least two distinct lineages (C1 and D) occurred in the Bumbuna areas. Further genetic and morphological studies are required to establish species boundaries and assign valid names.

Hipposideros aff. ruber C1 was represented by five samples in the genetic sequencing (Fig. 2), with four bats from this study, captured at four sites (B1, B4, B7, B8) in 2016. The two females were pregnant (1 + 7 Apr). The echolocation call frequencies of the individuals sampled had an unusually wide range for a hipposiderid bat (Table 5). Lineage C1 seems to occur in the forest zone of West and Central Africa, but additional data are needed to corroborate this assumption. Recently, the taxon was reported from Mount Nimba in Liberia (Monadjem et al. 2016).

Genetic sequencing revealed that 13 samples belonged to Hipposideros aff. ruber D. Eight of these bats were from six sites visited in this study (B1, B2, B4, B5, B6, Lo1). One of four females was pregnant (24 Mar) and one was lactating (2 Apr). Echolocation call frequencies of two males were higher than the frequencies used by four females (Table 5). Frequency differences between males and females have previously been observed in lineage D1 (Vallo et al. 2011). Hipposideros aff. ruber D is currently only known from forested areas in West Africa. The majority of our specimens groups with individuals from lineage D1 (Vallo et al. 2011). Three bats form a distinct branch on the phylogenetic tree as sister taxon to lineage D1 and might constitute another lineage (Fig. 2;Hauslaib-Haidn 2011, Herkt et al. 2016 Suppl. material).

***Hipposideros abae* J.A. Allen, 1917** Aba Leaf-nosed Bat

We captured two females of the Aba roundleaf bat at Kamin Mata (Ba) during the day, one was lactating (24 May). At the time of visit, the cave harboured a colony of roughly 100 individuals of this species and around 300 individuals of Hipposideros aff. ruber. Two males were caught around the transmission line (B5) near Kamin Mata, just on the other side of Seli River. The cf frequency of one female (Table 5) was recorded and appears to be the first recording of this species.

***Macronycteris vittatus* (Peters, 1852)** Striped Leaf-nosed Bat

*Macronycteris* has long been considered a synonym of *Hipposideros*. We follow Foley et al. (2017) in recognising the genus *Macronycteris* as paraphyletic to *Hipposideros*, based on genetic analyses using mitochondrial and nuclear DNA sequences as well as morphological diagnoses (Vallo et al. 2008, Monadjem et al. 2013, Foley et al. 2017).

**Figure 7.**
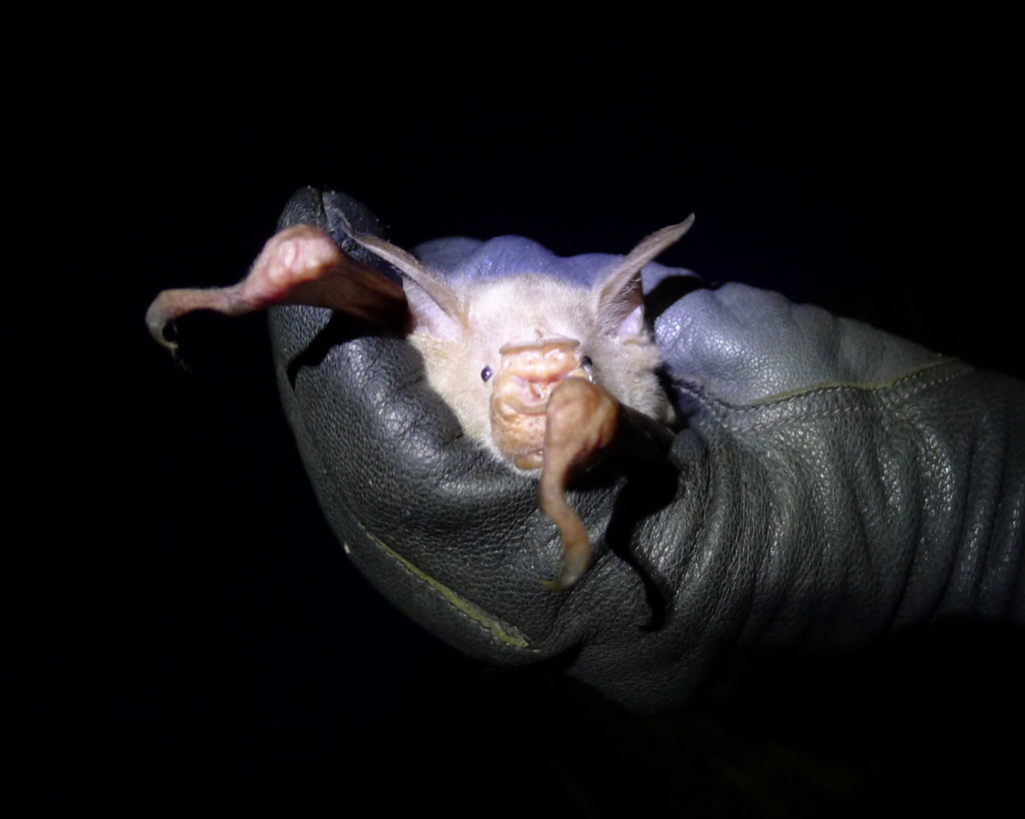
Head of female *Macronycteris vittatus* (RCJF-NW3664) recorded at the western outskirts of the Loma Mountains National Park, Sierra Leone.

We recorded two individuals of this large hipposiderid in 2016, one pregnant female (26 Mar; Fig. 7) in Loma and one sub-adult male in a canopy net across the Makarikari River (B8). Echolocation frequencies were well above 60 kHz (Table 5; Happold 2013b), which excludes the morphologically similar *M. gigas* that is known to use lower frequencies up to 56 kHz. The striped leaf-nosed bat or *M. gigas* was also observed in Bumbuna I, but the individual was not clearly identified (Decher et al. 2010). *Macronycteris vittatus* is listed as “Near Threatened” on the IUCN Red List (IUCN 2019). The records from our study areas represent the second and third confirmed localities for the species in Sierra Leone, the first one being from Fadugu near Bumbuna II (Grubb et al. 1998 [as *Hipposideros commersoni*]). *Macronycteris vittatus* has a scattered distribution in West Africa and its population is considered to be declining due to loss or disturbance of suitable habitat and hunting. The species depends at least partially on caves as day roost.

***Doryrhina cyclops* (Peters 1871)** Cyclops Leaf-nosed Bat

We follow Foley et al. (2017) in raising *Doryrhina* to genus level and recognising it as paraphyletic to *Hipposideros*, based on genetic analyses using mitochondrial and nuclear DNA sequences as well as morphological diagnoses (Vallo et al. 2008, Monadjem et al. 2013, Foley et al. 2017).

We captured four individuals of Cyclops leaf-nosed bat in 2014, two males and two females which were both lactating (26 + 28 May). One female was encountered at B3, the other three individuals were captured when emerging from their day roost, a hollow tree on a freshly burnt field at B2. Echolocation calls of the two females were recorded and frequencies were in the common range of *D. cyclops* (Table 5; Fahr 2013c).

**Family Nycteridae**

***Nycteris arge* Thomas, 1903** Bates's Slit-faced Bat

One male was captured in riparian forest at B1.

***Nycteris grandis* Peters, 1865** Large Slit-faced Bat

One male of the large slit-faced bat was recorded in wooded savannah at B6, representing the third record for Sierra Leone (Decher et al. 2010).

***Nycteris macrotis* Dobson, 1876** Large-eared Slit-faced Bat

We captured two males of this species, one in forest at B1 and one in degraded riparian forest at the Seli River (B4). The large-eared slit-faced bat is a widespread species, which has been previously documented from several other localities in Sierra Leone (Grubb et al. 1998). *Nycteris macrotis* occurs in a variety of habitats and it uses caves and hollow trees as day roost.

***Nycteris thebaica* E. Geoffroy, 1818** Egyptian Slit-faced Bat

Three individuals of *N. thebaica* were recorded in Bumbuna II, all in riparian forest at the cave system at B1, one male and one female in 2014, and one sub-adult female in 2016. *Nycteris thebaica* is difficult to distinguish from *N. gambiensis*, but the latter is smaller than *N. thebaica* in West Africa (Van Cakenberghe and De Vree 1998). Forearm lengths of our adult individuals were rather in the range of *N. thebaica* (42.4 and 43.1 mm). Fur colouration also points to *N. thebaica*, as all individuals had a much lighter ventral than dorsal fur. There are two previous record of *N. thebaica* from Sierra Leone, from Freetown and Mongberi near Bo (Van Cakenberghe and De Vree 1998). This species occurs predominantly in wooded savannahs in West Africa. It requires caves or cave-like structures as day roost.

***Nycteris hispida* (Schreber, 1775)** Hairy Slit-faced Bat

Four individuals were found roosting in an open cavity under a large rock in a partly dried out riverbed near B2 during the day. One adult and one sub-adult flew off, but one lactating female (25 May) was captured with a young attached. *Nycteris hispida* is known from other locations in the Northern Province (Van Cakenberghe and De Vree 1993), and it is generally found in different habitats from savannahs to forests.

**Family Emballonuridae**

***Coleura afra* (Peters, 1852)** African Sheath-tailed Bat

**Figure 8.**
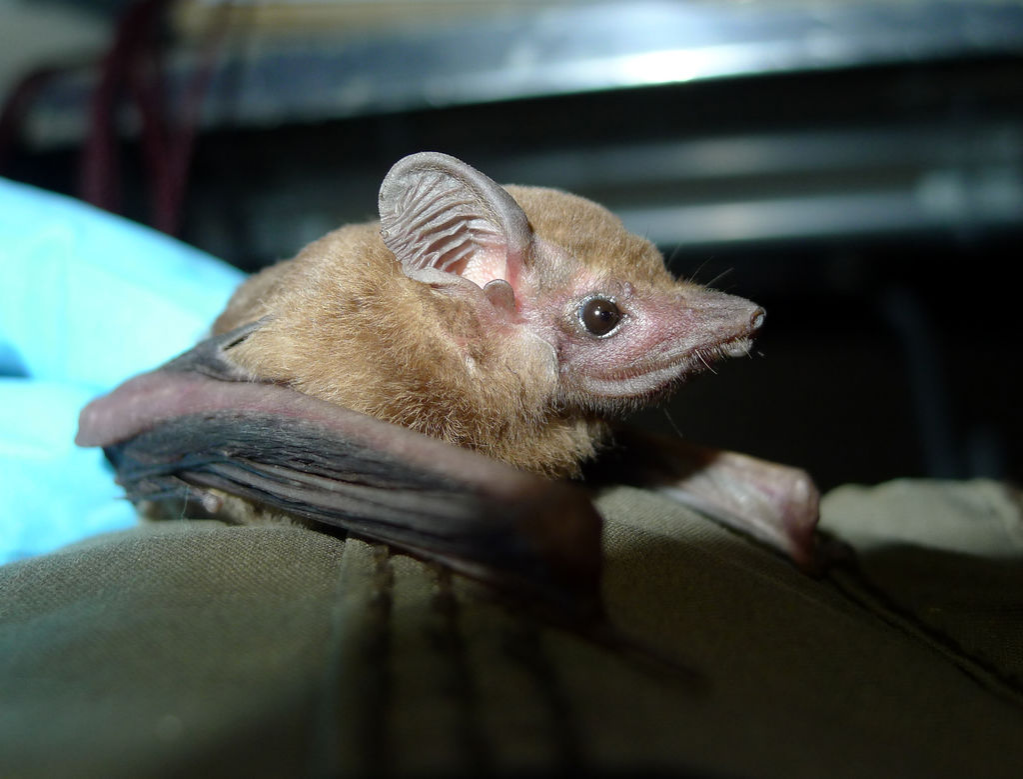
*Coleura afra* (RCJF-NW3790) recorded in the Bumbuna II Phase area in the Northern Province, Sierra Leone.

We captured one male of this emballonurid bat in riparian forest at B4 in 2016 (Fig. 8). This record of *C. afra* constitutes the first for Sierra Leone, alongside a colony comprising several hundred individuals observed on Bunce Island, a small island in the estuary of the Rokel River, in 2014 (N. Weber pers. observation; iNaturalist.org 2019). In West Africa, *Coleura afra* has a patchy distribution in savannah and savannah transition zones (Benin, Ghana, Guinea, Guinea-Bissau, Côte d'Ivoire, Nigeria, Togo; Happold 2013c). It requires caves or cave-like structures as day roost.

**Family Vespertilionidae**

***Myotis bocagii* (Peters, 1870)** Rufous Mouse-eared Bat

A total of seven individuals was obtained in both study periods. Six records were from the Seli River (B5, B6) and one individual was captured over Makarikari River (B8). The single female was pregnant (4 Jun).

***Pipistrellus nanulus* Thomas, 1904** Tiny Pipistrelle

We recorded one male and one female in 2014 (B4, B6). The tiny pipistrelle was previously documented from four localities in the country (Grubb et al. 1998).

***Scotoecus hirundo* (de Winton, 1899)** Dark-winged Lesser House Bat

Our capture represents the second record of *S. hirundo* for Sierra Leone, the first is from Musaia approximately 50 km north of our locality (Grubb et al. 1998). The female was captured over rocks in the Seli River near the access road (B6). This species seems to be mainly associated with savannah habitats.

***Scotophilus viridis* (Peters, 1852)** Green House Bat

We captured one male in the savannah landscape at B3. Grubb et al. (1998) mention two historic records from the north of Sierra Leone, which refer to *S. viridis* [as *S. nigritellus*]. Taxonomic relationships within the genus *Scotophilus* are currently not clear and need a revision (Vallo et al. 2016, Demos et al. 2018). The green house bat is associated with savannahs and woodlands.

***Scotophilus nux* Thomas, 1904** Nut-coloured Yellow Bat

One male was captured over a swamp at the forest edge in Loma. The nut-coloured yellow bat has been previously recorded from the rainforest zone in southern Sierra Leone. Our record constitutes the northernmost locality of this species to date, with the nearest record from 10 miles north of Panguma, 100 km to the south of Loma (Grubb et al. 1998 [as *S. dinganii nux*]). The distribution of *S. nux* in West Africa ranges from Sierra Leone to Ghana in the rainforest zone and continues eastwards in Nigeria.

***Scotophilus dinganii* (A. Smith, 1833)** Yellow-bellied House Bat

Our samples included two pregnant females (7 Apr, 29 May). We encountered one bat in woodland savannah (B3), and one over the Makarikari River (B8). The yellow-bellied house bat was previously known from three localities in Sierra Leone, Bonthe (Robbins et al. 1985), Kent (ROM 62749) and Njala (MRAC 35641, 35685). The species occurs mainly in woodlands and savannahs.

***Glauconycteris poensis* (Gray, 1842)** Abo Butterfly Bat

**Figure 9.**
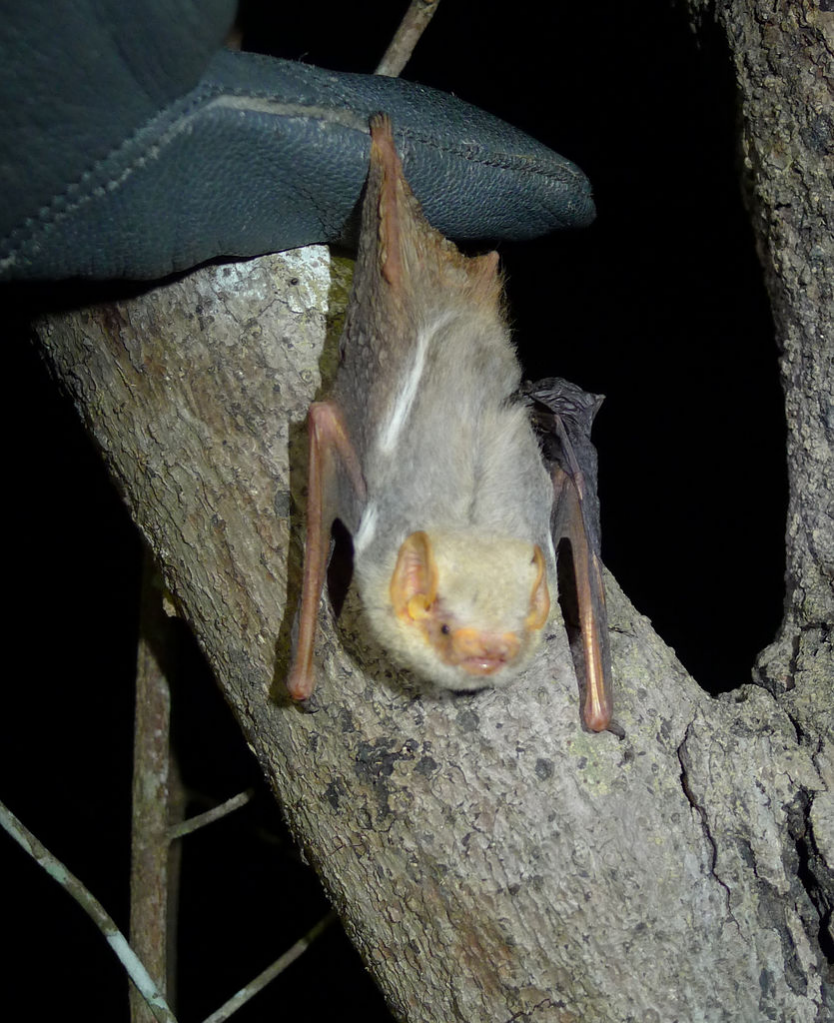
*Glauconycteris poensis* (NW2864) recorded in the Bumbuna Phase II area in the Northern Province, Sierra Leone.

We recorded a total of six males of this species in both study periods and areas. Four individuals were captured at B5 in 2014 after emergence from their day roost. One individual was caught over a small river at B7 and one individual was from Loma. *Glauconycteris poensis* is known from several localities in the south of Sierra Leone (Hayman and Jones 1950, Grubb et al. 1998). Fur colour of this bat is highly variable, indicating that the name *G. poensis* might generally refer to more than one species. All our specimens had a pale cream-brown to greyish colour, two whitish shoulder spots dorsally, and two whitish lateral bands dorsally, the latter being a typical character of the species in West Africa (Fig. 9).

**Family Molossidae**

***Chaerephon nigeriae nigeriae* Thomas, 1913** Nigerian Free-tailed Bat

We captured one nulliparous female in Loma, which constitutes the second record of this savannah species for Sierra Leone. The first record is from east of Fintonia in Outamba-Kilimi National Park (Grubb et al. 1998), approximately 120 km west-northwest from our site.

***Mops condylurus* (A. Smith, 1833)** Angolan Free-tailed Bat

Seven individuals of this molossid bat were captured with canopy nets, three in savannah habitats (B3), one over Makarikari River (B8) and three in Loma. There are several previous records of *M. condylurus* from Sierra Leone (Grubb et al. 1998). The species is widely distributed in a broad range of habitats including agricultural landscapes. Natural day roosts are in tree openings, but Angolan free-tailed bats also use roofs and other anthropogenic structures for roosting.

***Mops nanulus* J. A. Allen, 1917** Dwarf Free-tailed Bat

This very small molossid was only encountered in Loma. Of the six individuals, four were females, one being pregnant (26 Mar). Our record is the third of *M. nanulus* for the country (Grubb et al. 1998 [as *Tadarida nanula*]). The locality at the fringe of the Loma Mountains corresponds to previous findings, which suggest that this species is associated with forest and forest edge habitats.

***Mops thersites* (Thomas, 1903)** Railer Free-tailed Bat

A single female of this species was recorded in Loma. *Mops thersites* was previously documented from several localities in the southern half of the country (Rosevear 1965, Grubb et al. 1998 [as *Tadarida thersites*]). In general, this molossid bat occurs in forest habitats.

***Mops trevori* J.A. Allen, 1917** Trevor's Free-tailed Bat

**Figure 10.**
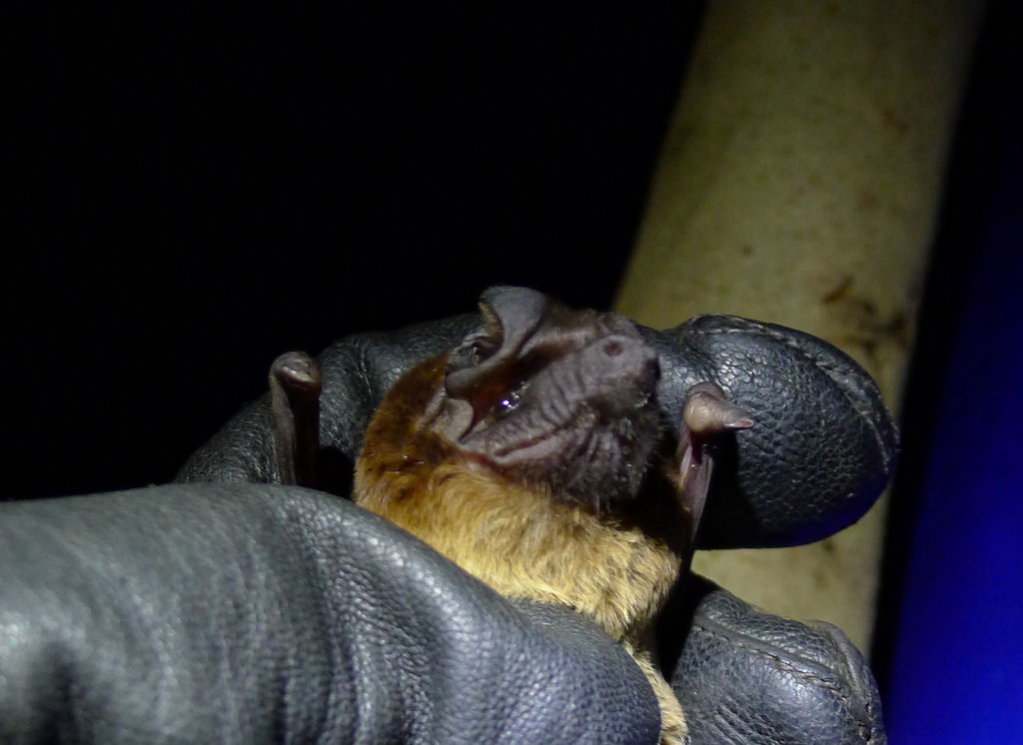
*Mops trevori* (NW3647) recorded in the Bumbuna Phase II area in the Northern Province, Sierra Leone.

We recorded two females of this rarely observed bat in Loma, one being pregnant (25 Mar; Fig. 10). *M. trevori* is documented for the first time from Sierra Leone. Our record constitutes the westernmost locality of this species, with a range extension of roughly 310 km. The nearest known site is in the Mount Béro Forest Reserve in southeastern Guinea (Fahr et al. 2006). *Mops trevori* is listed as “Data Deficient” by the IUCN Red List (IUCN 2019). It is known from a few disjunct records in forest-savannah mosaic habitats, with the easternmost in Uganda (Happold 2013d).

**Order Soricomorpha**

**Family Soricidae**

***Crocidura olivieri* (Lesson, 1827)** Olivier’s Shrew, African Giant Shrew

One individual of this large common shrew was captured in a pitfall trap close to the Seli River at B2. The species is found in a wide variety of habitats including forest, savannah, degraded forest, farmbush, shrubland and forest clearings in most of sub-Saharan Africa (Churchfield and Hutterer 2013). *Crocidura olivieri* is also often found near human settlements (IUCN 2019). There are numerous localities of this species in Sierra Leone (Grubb et al. 1998). Following Jacquet et al. (2015), our specimen belongs to clade I, which is associated with West African rain forests. Our individual was captured in microhabitat with no canopy cover.

**Crocidura cf. theresae (Heim de Balsac, 1968)** Therese’s Shrew

One male was captured in a pitfall trap at B9. This species was tentatively assigned to *C. theresae* based on morphological characteristics (head-body length: 89.0 mm, tail: 62.0 mm, hind foot: 15.0 mm, ear: 6.9 mm, body mass: 14.5 g). Molecular analyses supported another position within the *C. poensis* species group, *C. grandiceps* (Jacquet et al. 2012), but this species was ruled out due to significant morphological differences. There are several other localities of this species throughout Sierra Leone (Grubb et al. 1998). *Crocidura theresae* has been recorded from mixed forest, grassland and rice fields (Heim de Balsac 1968). It occurs in West Africa in the savannahs from Guinea to Ghana. Our individual was captured in microhabitat with 5% canopy cover.

***Crocidura* sp.1**

One pregnant female of this shrew was captured at B1 (22 May). This individual also belongs to the *C. poensis* species complex. Our specimen shares morphological characteristics with *C. longipes* (head-body length: 93.0 mm, tail: 59.0 mm, hind foot: 15.3 mm, ear: 7.3 mm, body mass: 19.0 g), but the genetic identification is not clear and requires further information and reference sequences. Our individual was captured in microhabitat with 75% canopy cover. The *C. poensis* species complex comprises large-sized species that are distributed throughout the Guinea-Congolian rainforests and savannahs. Taxonomic relationships within this group are currently not resolved and a revision based on morphological and genetic studies is urgently needed.

**Order Rodentia**

**Family Nesomyidae**

***Cricetomys gambianus* (Wroughton, 1910)** Gambian Giant Pouched Rat, Giant Rat

Seven individuals (2 males, 4 females, 1 unspecified) of this large rodent were captured in Tomahawk traps at all sites except for B3. All but two individuals were caught in forest vegetation, one female on a branch at about 2 m height. One female was lactating (28 May). Three voucher specimens were identified as *C. gambianus* based on morphological and molecular analyses. The species occurs in grassland, woodland and anthropogenic habitats in the northern savannahs of West and Central Africa (Monadjem et al. 2015) and throughout Sierra Leone (Grubb et al. 1998). Compared to the distribution range and genetic sequences in Olayemi et al. (2012), our specimens belong to clade III. Our individuals were captured in microhabitat with an average of 52% canopy cover.

**Family Muridae**

***Gerbilliscus kempi* (Wroughton, 1906)** Kemp's or Northern Savannah Gerbil

One male of this common West and Central African gerbil was obtained at B5 in savannah with elephant grass. This species can be distinguished from the less common *G. guineae*, which is restricted to northern Sierra Leone, by its shorter, untufted tail (Granjon et al. 2012). It is widespread in savannahs from southern Senegal to Sudan (Grubb et al. 1998 [as *Tatera kempii*], Volobouev et al. 2007, Monadjem et al. 2015) where it inhabits grass- and farmland. Our individual was captured in microhabitat with no canopy cover.

***Lophuromys sikapusi* (Temminck, 1853)** Rusty-bellied Brush-furred Rat

One male of this distinctive orange-bellied rat was captured in a valley with fields at B9 in a pitfall trap set in dense tall elephant grass. This individual was captured in microhabitat with no canopy cover.

***Uranomys ruddi* (Dollmann, 1909)** Rudd's Brush-furred Mouse

Only one female of this distinctive savannah and open woodland mouse was caught at the edge of a field at B9. The species is very rare in surveys of small mammals (Happold 2013) and in museum collections. *Uranomys ruddi* occurs from Senegal eastwards in the savannah zone to Zimbabwe and Mozambique, preferably in savannahs with ravine or similar forests (Denys et al. 2009). This widespread but patchily distributed taxon most likely includes several distinct but morphologically cryptic species. This individual was captured in microhabitat with no canopy cover.

***Hylomyscus simus* (Allen and Coolidge, 1930)** West African Wood Mouse

One male of this arboreal species was caught on a branch in young forest at B5. We follow Nicolas et al. (2006) in using *H. simus* for the common West African form. The potentially sympatric species *H. baeri* can be distinguished from *H. simus* by its pure white ventral pelage. The one individual was captured in microhabitat with 90.0% canopy cover.

***Lemniscomys striatus* (Linnaeus, 1758)** Typical Striped Grass Mouse

Seven individuals (2 male, 2 female, 3 unspecified) of this striped grass mouse were caught at three sites, mostly in savannahs with elephant grass (B5, B9) or termite mounds (B3). One female was pregnant with 4 embryos (30 May). Following Nicolas et al. (2008), our specimens belong to their clade IV, covering West Africa from Guinea to Ghana. All individuals were captured in microhabitat with an average of 6.4% canopy cover.

***Malacomys edwardsi* (Rochebrunne, 1885)** Edward's Swamp Rat

One male was caught on a steep hill in gallery forest at B1 and one pregnant female with two embryos in young forest near the Seli River at B6. Both individuals were captured in microhabitat with an average of 93.5% canopy cover.

***Mastomys erythroleucus* (Temminck, 1853)** Multimammate Mouse

Seven specimens of this common rodent were captured at all study sites except for B1 and B9. Two males were captured at B2 in a rice patch near a palm oil cooking site, one male at B3 in elephant grass savannah, three (two females, one unspecified) at B6 in grassland in a marshy area near the shore of the Seli River and one male at B5. Our specimens belong to the West African phylogroup A (Brouat et al. 2009) and were captured in microhabitat with an average of 1.7% canopy cover.

***Mus musculoides* (Temminck, 1853) / *M. minutoides* (Smith, 1834)** Pigmy Mice

One male belonging to this species complex was captured at B2. It is distinguished from *M. setulosus* based on its small size. This tiny mouse was captured in microhabitat with 40.0% canopy cover.

***Mus setulosus* (Peters, 1876)** Peter's Pygmy Mouse

One male of this relatively large pygmy mouse species was captured at B1 in riparian forest and three males at B9 in elephant grass and shrubs. All individuals were captured in microhabitat with an average of 25.0% canopy cover.

***Praomys rostratus* (Miller, 1900)** West African Soft-furred Mouse

We captured 71 individuals (21 male, 36 female, 14 unspecified) of this common forest rat. It was the most common rodent species recorded in our study and captured at almost all study sites (B1, B2, B3, B6, B9), almost exclusively in forest habitats. Seven females were pregnant (one with two embryos, 24 May; two with three embryos, 23 + 27 May). The microhabitat of 68 captured individuals had an average of 73.4% canopy cover.

**Family Sciuridae**

***Paraxerus poensis* (Smith, 1834)** Green Bush Squirrel

One individual was photographed on 3 June by ornithologist Paul Robinson at B3. This squirrel is common in forest edge, secondary forest and farmbush habitats. It is widely distributed in the Upper Guinean Forests of West Africa to the River Volta and in the Lower Guinean Forests from the Niger River eastwards into Central Africa (Emmons 2013).

***Euxerus erythropus* (Geoffroy, 1803)** Striped Ground Squirrel

Observed but not photographed crossing the road at S3 on 20 May.

## Discussion and conclusion

Our study shows that Bumbuna II and its surroundings harbour a species-rich small mammal fauna and highlights the relevance of biodiversity surveys in understudied areas for impact assessments prior to major development projects. We documented 30 bat species from Bumbuna II, half of which were not previously known from the wider area (Table 2), and added three species to the list of Sierra Leone, while another three species are second records for the country. Four bat species encountered in Bumbuna II are of global conservation concern. We recorded three shrew and eleven rodent species, with all shrews and three rodents constituting new records for the area.

The species accumulation curves for bats rise continuously, suggesting that additional species can be expected (Fig. 4). Combining our and previous records, 37 bat species are known within a < 40 km radius around Bumbuna II. These together with our records from Loma, at a distance of slightly more than 40 km east-southeast from Bumbuna II, yield a total of 42 bat species for the corresponding area. These figures are in the range of the species richness predicted for Bumbuna II by the three estimators (38-45 species). The number of bat species recorded in Bumbuna II surpasses the species totals obtained in single or rapid assessments in the region (Liberia and Guinea: 7-25 species; Fahr et al. 2006, Monadjem and Fahr 2007, Weber and Fahr 2007), and is in the range of studies with repeated sampling (Guinea and Côte d'Ivoire: 30-40 species; Fahr et al. 2006, Fahr and Kalko 2011, Decher et al. 2015). Our records from Loma provide only a first insight into the bat fauna of this mountain area, as sampling was limited to four nights at one site. With 15 species encountered in a very short period, we assume that considerably more species occur in the Loma Mountains and further surveys should be conducted.

We raised the number of bat species documented from Sierra Leone from 58 to 61. The 30 bat species observed in Bumbuna II within an area of 82 km^2^ represent almost half of the 61 species known to occur in Sierra Leone (49.2%). The 42 bat species recorded in a wider area spanning 2,220 km^2^, which corresponds to 3% of the country surface (71,740 km^2^), represent more than two thirds of the national species pool (68.9%). These figures suggest that the assessment of bats on a national scale is also not yet complete.

The diverse habitat mosaic of Bumbuna II was reflected in the composition of bat species, with similar proportions of savannah (36.7%) and forest (43.3%) bat species, the latter being slightly dominant. However, the presence of bat species differed between seasons. For instance, the proportion of fruit bats in overall captures varied greatly, with 18.5% in three species at the onset of the wet season (2014) and 84.2% in eight species in the dry season (2016). This is probably driven by seasonal and spatial distribution of resources, while interannual variability might also play a role.

Bumbuna II harboured a high proportion of obligatorily and partially cave-roosting bats (Table 2; n = 13, 43.3%), which was even slightly higher for both Bumbuna areas combined (n = 17, 45.9%). The distribution of caves is spatially uneven and constitutes a limiting habitat element for cave-dwelling bats, which often have a patchy and/or range-restricted distribution. Accordingly, five of six species documented from the Bumbuna areas and listed in a threatened category of the IUCN Red List are associated with caves.

The species accumulation curve for terrestrial small mammals rises continuously and does not start to level off (Fig. 5), indicating that the inventory of small terrestrial mammals in Bumbuna II is also not yet complete. The trapping effort was below the minimum of 409-500 trap nights per site, which was recommended for preliminary inventories for an environmental impact assessment (Jones et al. 1996, Fraser et al. 2003). The trap success of our study (7.1%) was in the range of other studies in the region (3.2-9.4%; Barnett et al. 2000, O'Brien et al. 2006, Decher et al. 2010). The number of species was high for the short sampling period compared to other studies with more trap nights (Barnett et al. 2000: 10 rodent species in 4,315 trap nights; Fichet-Calvet et al. 2009: 14 species in 3,465 trap nights; Fichet-Calvet et al. 2010: 17 species in 12,784 trap nights; Konečný et al. 2010: 11 species in 5,400 trap nights). The findings of Decher et al. (2010) and our study, with six new records for the area, result in a total of 19 (6 shrew and 13 rodent) species for both Bumbuna areas, which is below the estimated richness for Bumbuna II (21-27 species). The commensal *Rattus rattus, Mus musculus* and *Mastomys natalensis* have been reported within < 40 km distance from Bumbuna II (Grubb et al. 1998) and might occur there, adding up to a total of 22 species, but the presence of species not previously recorded from the area is also likely.

The high proportion of savannah species (46.2%) contrasts the results for bats, and might result from over-proportional sampling of savannah habitat and low mobility of terrestrial small mammals. However, three species associated with savannah (*C. gambianus, G. kempi, U. ruddi)* were only found in Bumbuna II, and three forest-dependent species (*C. jouvenetae, C. emini, H. planifrons)* only in Bumbuna I. The forest species *Praomys rostratus* was the most common terrestrial small mammal species in Bumbuna II, as in Bumbuna I (Decher et al. 2010) and other studies in the Upper Guinean Forests (Denys et al. 2009, Fichet-Calvet et al. 2010). No predominantly commensal or invasive species was encountered during our survey, pointing to a rather undisturbed species assemblage.

*Lophuromys sikapusi* was rare in Bumbuna II (beginning of wet season), but common in Bumbuna I (beginning of dry season). This might support that *L. sikapusi* does not reproduce during the dry season as has been suggested by Fichet-Calvet et al. (2009). Seasonal abundance patterns of other small mammal species might have similarly influenced the results of our study, as has been shown elsewhere (Attuquayefio and Wuver 2003, Nicolas and Colyn 2003, Makundi et al. 2005, Habtamu and Bekele 2013).

The combined findings from Bumbuna II and I underline that high habitat heterogeneity in the transition zone between forests and savannahs in West Africa supports both forest-dependent and savannah species and fosters species richness, as has been previously reported for bats (Fahr and Kalko 2011). The occurrence of at least six bat species of global conservation concern in the Bumbuna areas confirms the importance of mountainous habitats in the region for rare and/or endemic bat species (Fahr et al. 2006, Weber and Fahr 2007, Decher et al. 2015, Monadjem et al. 2016). Knowledge on several small mammal species recorded in this study is rudimentary, and some species have not yet been assessed. At the same time, mountainous forest habitats in the Upper Guinean Forests are of priority for extractive and logging industries, and under additional pressure through agricultural expansion. Economic development in these areas in Sierra Leone is to be carefully considered against negative environmental effects that impact biodiversity, ultimately also affecting welfare of the human population. The two Bumbuna areas are situated along the same river approximately 30 km apart and should be perceived as one continuous landscape. Flooding of the Seli River for the second Bumbuna dam adds to cumulative environmental impacts in the area, in particular the loss of ecologically valuable habitats. For instance, the nearby Sula Mountains are currently the target of several mining projects (industrial and small-scale). The largest seasonal freshwater lake in the country, Lake Sonfon, is located there, and already strongly marked by human impacts despite being proposed as a national park, supported by national conservation initiatives (Sesay et al. 2017). In order to preserve biodiversity in this region and offset environmental impacts from the second Bumbuna dam, we endorse the establishment of an adequate conservation area, similar to the designation of the Loma Mountains National Park for the Bumbuna Phase I dam. This could potentially involve the Sula Mountains and Lake Sonfon.

## Supplementary Material

Supplementary material 1 Microhabitat terrestrial small mammals Data type: Table and figure
Brief description: Microhabitat: proportion of ground cover types for terrestrial small mammals captured in Bumbuna II in 2014, based on percentage estimates at each trap (ca. 1x1m).
File: oo_273464.xlsx Ricarda Wistuba

Supplementary material 2 Rarefaction curves bats Data type: Table
File: oo_302742.xlsx Natalie Weber

Supplementary material 3 Rarefaction curve terrestrial small mammals Data type: Table
File: oo_273465.xlsx Ricarda Wistuba

Supplementary material 4 Gazetteer of localities Data type: Table
File: oo_302743.xlsx Natalie Weber

## Figures and Tables

**Figure 1a.**
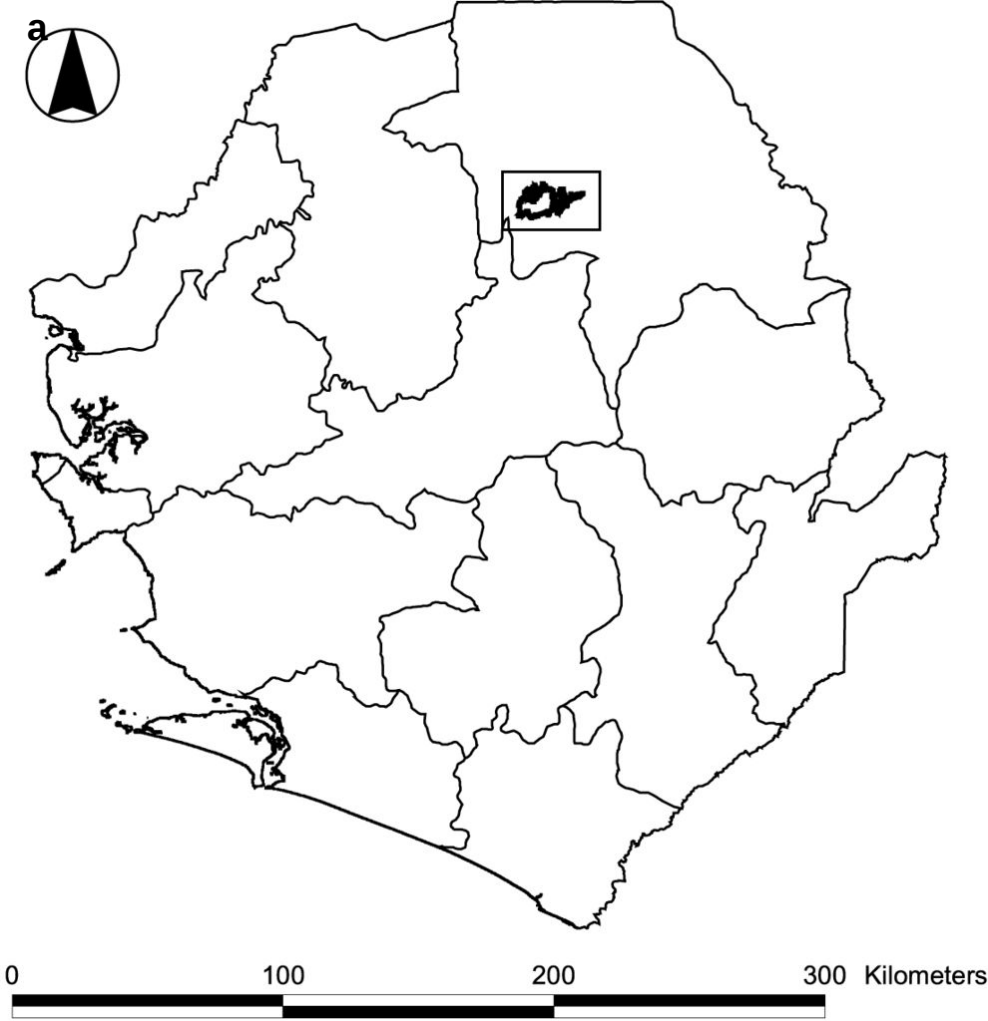
Outline map of Sierra Leone with district boundaries and the Bumbuna Phase II area indicated by rectangle

**Figure 1b.**
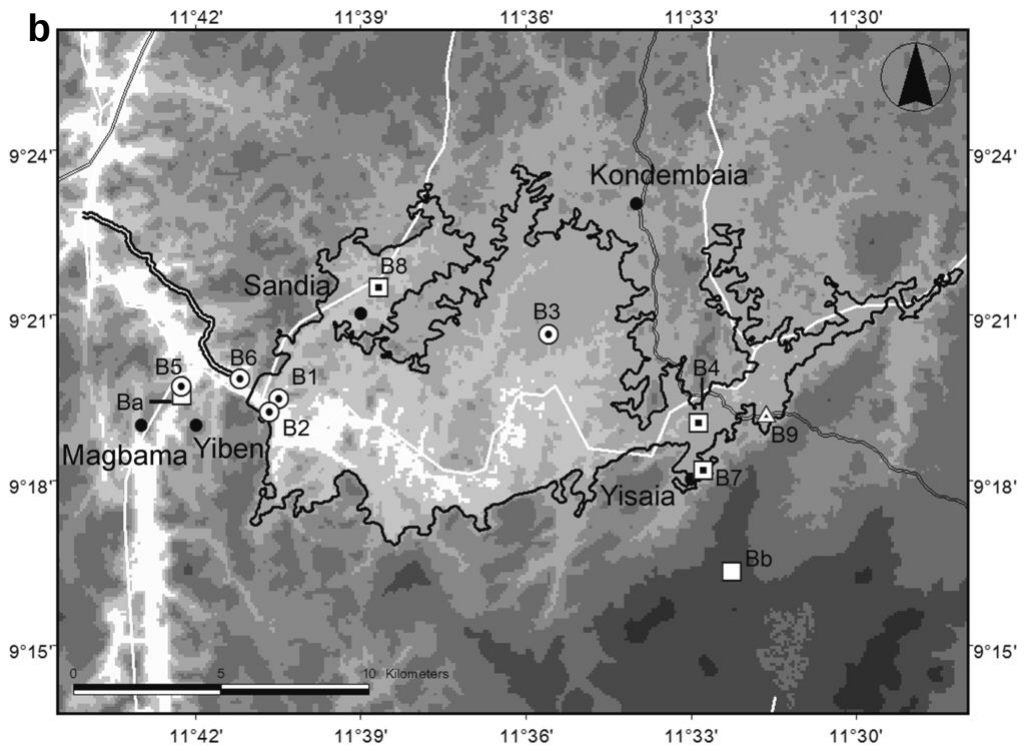
Study sites in Bumbuna II on SRTM 90 m digital elevation data (http://srtm.csi.cgiar.org; Jarvis et al. 2008). Dotted circle: bats and terrestrial small mammals, dotted square: only bats, dotted triangle: only terrestrial small mammals, white squares: caves used by bats. Black outline: Bumbuna II reservoir area, white lines: rivers, framed line: access road, thinly framed lines: roads.

**Figure 4a.**
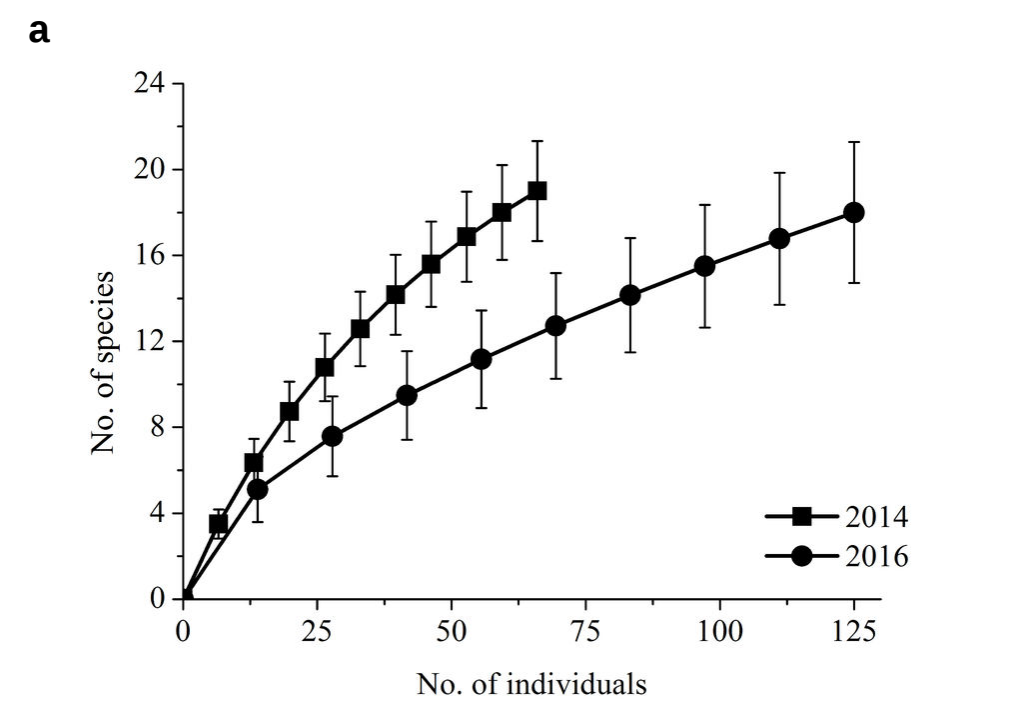
during surveys in 2014 and 2016

**Figure 4b.**
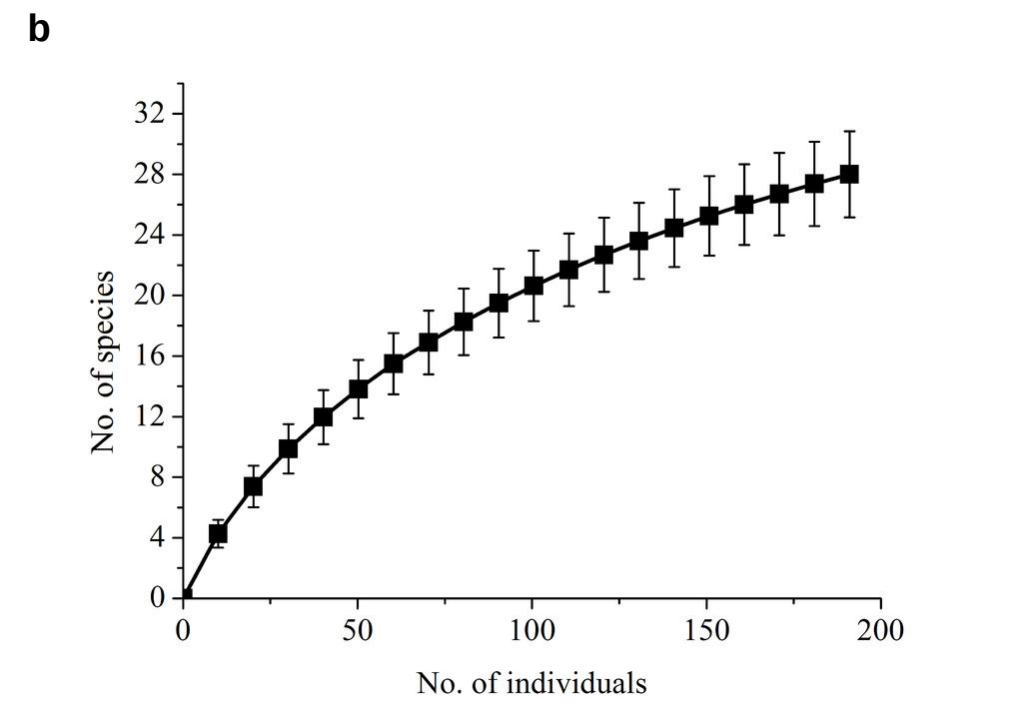
combined for both surveys

**Table 1.** Site IDs used in the text, full name, sampling year, coordinates and description of all study sites visited in the Bumbuna Phase II area (B) in the Northern Province, Sierra Leone, in 2014 and 2016 and at the western outskirts of the Loma Mountains National Park (Lo) in 2016.

**Site ID**	**Site Name**	**2014**	**2016**	**Coordinates**	**Description**
B1	Makarikari River 1	X	X	09°19'28.0"N, 11°40'29.8"W	Riparian forest, steep and rocky ravine at seasonal tributary, bordering savannah and palm plantation.
B2	Megboke River	X		09°19'13.3"N, 11°40'39.8"W	Riparian forest at rocky tributary, farmbush with palm plantation, secondary forest on slope, village.
B3	Pallama	X	X	09°20'37.2"N, 11°35'35.2"W	Hilly savannah, elephant grass, stony ground, xerophyte trees, patches of riparian forest and farmland.
B4	Yogoron River 1	X	X	09°19'01.3"N, 11°32'52.3"W	Mouth of Yogoron River, riparian forest, mangrove elements, sandbank with vegetation and rocks.
B5	Makpoton River	X		09°19'41.1"N, 11°42'15.7"W	Savannah, elephant grass on plateau, creeks, riparian forest, palm field. Degraded bare dry soil at shore.
B6	Access road	X		09°19'49.4"N, 11°41'11.8"W	Farmbush, steep slope, creek, young riparian forest; marsh areas, dead trees, sandy beach and bushes.
B7	Yogoron River 2		X	09°18'09.8"N, 11°32'46.9"W	Riparian forest, agricultural and swamp lands; forested hills, artisanal gold mining.
B8	Makarikari River 2		X	09°21'27.6"N, 11°38'41.2"W	Riparian forest, river with rocky steps, small sandy tributary.
B9	Badala	X		9°19'12.18"N, 11°31'36.84"W	Swamp area, peanut and rice fields, small river, bushy vegetation with tall elephant grass, village, forest patches.
Ba	Kamin Mata Cave	X		09°19'31.4"N, 11°42'16.0"W	Accessible cave 10-15 m length, heavily degraded surroundings.
Bb	Yafarama Cave		X	09°16'18.8"N, 11°32'16.6"W	Partly accessible cave, two openings, underneath plateau in hilly wood- and farmland.
Lo1	Dorro River		X	09°13'31.5"N, 11°11'53.3"W	Patches of forest, farmbush, cultivated and plantation land, swamp at river.
Loa	Sadia Konkoma Cave		X	09°14'02.0"N, 11°12'21.3"W	Steep and rocky forested slope, several cavities, vegetation damaged by recent fire.

**Table 2.** Bat species recorded in the Bumbuna Phase II area and west of the Loma Mountains National Park (Loma) during this study, in the Bumbuna Phase I area (Decher et al. 2010), and previous references within 40 km of Bumbuna II or, in brackets, Loma. Habitat: coarse assignment to preferred habitat types (F: forest, S: savannahs and woodlands, in brackets: marginally including respective habitat type). Cave: strict “+” or facultative “(+)” dependency on caves as day roosts. RL: international Red List status (EN: Endangered, VU: Vulnerable, NT: Near Threatened, DD: data deficient, n.a.: not assessed, all species without entry: Least Concern, IUCN 2019). B: Bumbuna areas, SL: Sierra Leone. *: only observed, not captured. For most small terrestrial mammals, previous localities are only approximate and might slightly exceed < 40 km radius around Bumbuna II. ^1^: Vallo et al. 2008, ^2^: Foley et al. 2017, ^3^: as *Mastomys hildebrandtii* .

**Family or (sub-)order / Species**	**BII 2016**	**BII 2014**	**BI 2006**	**Loma 2016**	**Previous references**	**RL**	**Habitat**	**Cave**	**Record**
** Pteropodidae **									
*Micropteropus pusillus*	X	X	X		Bergmans 1989		S		
* Epomops * * buettikoferi *	X	X	X	X			F(S)		
*Eidolon helvum*	X			X		NT	FS		1st B
*Hypsignathus monstrosus*	X			X*	Atkinson et al. 1996, Grubb et al. 1998		F(S)		1st B
*Rousettus aegyptiacus*	X		X	X			FS	+	
* Myonycteris * * leptodon *	X	X	X				F(S)		
*Myonycteris angolensis*	X			X			F(S)	(+)	1st B
* Nanonycteris * * veldkampii *	X		X	X			F(S)		
** Rhinolophidae **									
*Rhinolophus landeri*		X			Grubb et al. 1998		(F)S	+	1st B
*Rhinolophus guineensis*	X		X			VU	F	+	
* Rhinolophus * * fumigatus *	X*		X				S	+	
*Rhinolophus denti* *knorri*			X				S	+	
*Rhinolophus ziama*			X			EN	F	+	
** Hipposideridae **									
*Hipposideros marisae*		X				VU	F	+	1st SL
*Hipposideros* aff. *ruber* C1^1^	X	?	X			n.a.	FS	(+)	
*Hipposideros* aff. *ruber* D^1^	X	X	X	X		n.a.	FS	(+)	
*Hipposideros jonesi*			X			NT	FS	+	
*Hipposideros abae*		X	X				S	+	
*Macronycteris vittatus* ^2^	X		?	X	Grubb et al. 1998	NT	S	(+)	2nd SL
*Doryrhina cyclops* ^2^		X	X				F(S)		
** Nycteridae **									
*Nycteris arge*		X	X				F		
*Nycteris grandis*		X	X				F		
*Nycteris macrotis*		X					FS	(+)	1st B
*Nycteris thebaica*	X	X					S	+	1st B
*Nycteris hispida*		X			Van Cakenberghe and De Vree 1993		FS		
** Emballonuridae **									
*Coleura afra*	X						S	+	1st SL
** Miniopteridae **									
*Miniopterus* [*schreibersii*] *villiersi*			X			n.a.	F(S)	+	
** Vespertilionidae **									
*Myotis bocagii*	X	X	X				F(S)		
*Pipistrellus inexspectatus*					Grubb et al. 1998		S		
*Pipistrellus nanulus*		X					F(S)		1st B
*Neoromicia somalica*					USNM 462850		S		
*Neoromicia* sp. nov. [aff. nana]			X			n.a.	FS		
*Scotoecus hirundo*		X					S		2nd SL
*Scotophilus viridis*		X					S		1st B
*Scotophilus nux*				X			F		
*Scotophilus dinganii*	X	X					S		1st B
*Glauconycteris poensis*	X	X		X			F		1st B
** Molossidae **									
*Chaerephon nigeriae*				X			S		2nd SL
*Mops condylurus*	X	X		X	USNM 462857		S		
*Mops nanulus*				X			F		
*Mops thersites*				X			F		
*Mops trevori*				X		DD	(F)S		1st SL
**Species total**	18/19*	**20**	**19**	14/15*					
**Species total BII**									**30**
**Species total BI and II, Loma and wider area**									**42**
** Soricomorpha **									
* Crocidura * *jouvenetae*			X				F		
*Crocidura nigeriae*			X				FS		
*Crocidura olivieri*		X			Grubb et al. 1998		FS		1st B
*Crocidura poensis*			X		Grubb et al. 1998		FS		
*Crocidura* cf. *theresae*		X			Grubb et al. 1998		FS		1st B
*Crocidura* sp.1		X							1st B
** Rodentia **									
*Cricetomys emini*			X		Grubb et al. 1998		F		
* Cricetomys * * gambianus *		X			Grubb et al. 1998		S		1st B
*Gerbilliscus kempi*		X			Grubb et al. 1998		S		1st B
*Hybomys planifrons*			X				F		
*Hylomyscus simus*		X	X				F		
* Lemniscomys * * striatus *		X	X		Grubb et al. 1998		S		
* Lophuromys * * sikapusi *		X	X		Grubb et al. 1998		FS		
* Malacomys * * edwardsi *		X	X				F		
* Mastomys * * erythroleucus *		X	X		Grubb et al. 1998		S		
* Mastomys * * natalensis * ^3^					Grubb et al. 1998		S		
*Mus musculus*					Grubb et al. 1998				
*Mus musculoides*/ *minutoides*		X	X		Grubb et al. 1998		S		
*Mus setulosus*		X	X		Grubb et al. 1998		FS		
*Praomys rostratus*		X	X		Grubb et al. 1998		F		
*Rattus rattus*					Grubb et al. 1998				
*Uranomys ruddi*		X			Grubb et al. 1998		S		1st B
**Species total**		**14**	**13**						
**Species total BI and II and wider area**									**22**

**Table 3.** Bats recorded at eight study sites and two caves in the Bumbuna Phase II area (B) in 2014 and 2016, and at the western outskirts of the Loma Mountains National Park (Lo) in 2016. Study sites in italics were sampled in 2014 and 2016. *: only observed, not captured. Species totals treat Hipposideros aff. ruber as one species. ^1^ Vallo et al. 2008, ^2^ Foley et al. 2017.

**Family / Species**	***B1***	**B2**	***B3***	***B4***	**B5**	**B6**	**B7**	**B8**	**Ba**	**Bb**	**Lo1**	**Loa**	**Total**
** Pteropodidae **													
*Micropteropus pusillus*			10	4			7	6					27
*Epomops buettikoferi*	X*	X*	5	18		2	12	8			4		49
*Eidolon helvum*							2				2		4
*Hypsignathus monstrosus*							1				X*		1
*Rousettus aegyptiacus*				1				4		X*	12	X*	17
*Myonycteris leptodon*	1		1					4					6
*Myonycteris angolensis*			1								5		6
*Nanonycteris veldkampii*			1	13			28	8			44		94
** Rhinolophidae **													
*Rhinolophus landeri*	2												2
*Rhinolophus guineensis*							1						1
*Rhinolophus fumigatus*										X*			-
** Hipposideridae **													
*Hipposideros marisae*	5		1										6
Hipposideros aff. ruber	44	3	1	4	12		3	1	2	X*			70
Hipposideros aff. ruber C1^1^	1			1			1	1					4
Hipposideros aff. ruber D^1^	2	1		2	1	1					1		8
*Hipposideros abae*					2				2				4
*Macronycteris vittatus* ^2^								1			1		2
*Doryrhina cyclops* ^2^		3	1										4
** Nycteridae **													
*Nycteris arge*	1												1
*Nycteris grandis*						1							1
*Nycteris macrotis*	1			1									2
*Nycteris thebaica*	3												3
*Nycteris hispida*		2											2
** Emballonuridae **													
*Coleura afra*				1									1
** Vespertilionidae **													
*Myotis bocagii*					1	5		1					7
*Pipistrellus nanulus*				1		1							2
*Scotoecus hirundo*						1							1
*Scotophilus viridis*			1										1
*Scotophilus nux*											1		1
*Scotophilus dinganii*			1					1					2
*Glauconycteris poensis*					4		1				1		6
** Molossidae **													
*Chaerephon nigeriae*											1		1
*Mops condylurus*			3					1			3		7
*Mops nanulus*											6		6
*Mops thersites*											1		1
*Mops trevori*											2		2
**Total**	**60**	**9**	**26**	**46**	**20**	**11**	**56**	**36**	**4**	-	**84**	-	**352**
**Number of species**	**7** +1*	**3** +1*	**11**	**8**	**4**	**6**	**8**	**10**	**2**	3*	**14** +1*	1*	**34**

**Table 4.** Terrestrial small mammals recorded at six study sites in the Bumbuna Phase II area (B) in 2014, and capture effort and success.

**(Sub-)Order / Species**	**B1**	**B2**	**B3**	**B5**	**B6**	**B9**	**Total**
** Soricomorpha **							
*Crocidura olivieri*		1					1
Crocidura cf. theresae						1	1
*Crocidura* sp.1	1						1
** Rodentia **							
*Cricetomys gambianus*	1	1		1	2	2	7
*Gerbilliscus kempi*				1			1
*Hylomyscus simus*				1			1
*Lemniscomys striatus*			2	1		4	7
*Lophuromys sikapusi*						1	1
*Malacomys edwardsi*	1				1		2
*Mastomys erythroleucus*		2	1	1	3		7
*Mus musculoide/minutoides*		1					1
*Mus setulosus*	1					3	4
*Praomys rostratus*	40	16	4		9	2	71
*Uranomys ruddi*						1	1
**Total**	**44**	**21**	**7**	**5**	**15**	**14**	**106**
**Number of species**	**5**	**5**	**3**	**5**	**4**	**7**	**14**
Number of traps	115	122	122	72	89	122	
Trap nights	345	244	244	72	152	366	1423
**Trap success (%)**	**12.8**	**8.6**	**2.9**	**6.9**	**9.9**	**3.8**	**7.4**

**Table 5.** Echolocation calls of rhinolophid and hipposiderid bats recorded in the Bumbuna Phase II area 2014 and 2016, and at the western outskirts of the Loma Mountains National Park in 2016. Mean cf frequencies are given with standard deviation (SD). Min / Max: minimum / maximum frequency, n: sample size, M: male, F: female.

**Species**	**Mean±1 SD [kHz**]	**Min–Max [kHz**]	**n**	**Sex**
*Rhinolophus landeri*	102.0	101.5‒102.5	2	M
*Rhinolophus guineensis*	82.0		1	F
*Hipposideros marisae*	142.5	141.0‒144.0	2	F
*Hipposideros marisae*	146.5	146.0‒147.0	2	M
Hipposideros aff. ruber C1	145.7±4.5	142.0‒152.0	3	M/F
Hipposideros aff. ruber D	122.5±2.4	120.0‒125.0	4	F
Hipposideros aff. ruber D	127.0		2	M
*Hipposideros abae*	103.0		1	F
*Macronycteris vittatus*	62.5	62.0‒63.0	2	M/F
*Doryrhina cyclops*	54.5	54.0‒55.0	2	F
*Rhinolophus fumigatus* (in flight, in Bb)	51.5		1	
